# Gene expression profiling in acute allograft rejection: challenging the immunologic constant of rejection hypothesis

**DOI:** 10.1186/1479-5876-9-174

**Published:** 2011-10-12

**Authors:** Tara L Spivey, Lorenzo Uccellini, Maria Libera Ascierto, Gabriele Zoppoli, Valeria De Giorgi, Lucia Gemma Delogu, Alyson M Engle, Jaime M Thomas, Ena Wang, Francesco M Marincola, Davide Bedognetti

**Affiliations:** 1 Infectious Disease and Immunogenetics Section (IDIS), Department of Transfusion Medicine, Clinical Center and trans-NIH Center for Human Immunology (CHI), National Institutes of Health, Bethesda, Maryland, 20892, USA; 2 Clinical Research Training Program (CRTP), National Institutes of Health, Bethesda, Maryland, 20892, USA; 3 Rush University Medical Center, Rush Medical College, Chicago, Illinois, 60612, USA; 4 Luigi Sacco Hospital, Via G.B. Grassi, 20157 Milano, Italy; 5 Department of Internal Medicine (DiMI), University of Genoa, Viale Benedetto XV, 6, 16132 Genoa, Italy; 6 Center of Excellence for Biomedical Research (CEBR), University of Genoa, Viale Benedetto XV, 6, 16132 Genoa, Italy; 7 Laboratory of Molecular Pharmacology, Center for Cancer Research, National Cancer Institute, National Institutes of Health, Bethesda Maryland, 20892, USA; 8 University of Sassari, Department of Drug Sciences, Via Muroni 23 A, 07100, Sassari, Italy; 9 Department of Oncology, Biology, and Genetics, University of Genoa, and National Cancer Research Institute, Largo Rosanna Benzi 10, 16132 Genoa, Italy

## Abstract

In humans, the role and relationship between molecular pathways that lead to tissue destruction during acute allograft rejection are not fully understood. Based on studies conducted in humans, we recently hypothesized that different immune-mediated tissue destruction processes (i.e. cancer, infection, autoimmunity) share common convergent final mechanisms. We called this phenomenon the "Immunologic Constant of Rejection (ICR)." The elements of the ICR include molecular pathways that are consistently described through different immune-mediated tissue destruction processes and demonstrate the activation of interferon-stimulated genes (ISGs), the recruitment of cytotoxic immune cells (primarily through CXCR3/CCR5 ligand pathways), and the activation of immune effector function genes (IEF genes; granzymes A/B, perforin, etc.).

Here, we challenge the ICR hypothesis by using a meta-analytical approach and systematically reviewing microarray studies evaluating gene expression on tissue biopsies during acute allograft rejection. We found the pillars of the ICR consistently present among the studies reviewed, despite implicit heterogeneity.

Additionally, we provide a descriptive mechanistic overview of acute allograft rejection by describing those molecular pathways most frequently encountered and thereby thought to be most significant. The biological role of the following molecular pathways is described: IFN-γ, CXCR3/CCR5 ligand, IEF genes, TNF-α, IL-10, IRF-1/STAT-1, and complement pathways. The role of NK cell, B cell and T-regulatory cell signatures are also addressed.

## Introduction

Defining the interplay between molecular pathways within highly complex biological systems, such as those between immune cell networks and target tissues, is certainly a daunting task. The advent of high-throughput gene expression technology has served as an extremely useful tool to enable investigators to characterize biological events taking place within humans, reducing the inherent bias often generated by testing specific but restricted hypotheses derived from animal models. Previously, we applied this approach to profiling tumor lesions in humans, before and after immunotherapy, to identify molecular pathways activated during immune-mediated tumor rejection. These pathways illustrate a process characterized by the coordinated activation of interferon stimulated genes (ISGs), the recruitment of cytotoxic cells through the massive production of specific chemokine ligands, and the activation of immune effector function (IEF) genes (genes expressed by NK cells and CD8 T cells upon activation) [1-4]. Similar pathways have been described among other immune-mediated tissue destruction processes such as those occurring during autoimmunity, graft versus host disease (GVHD), infection clearance, acute cardiovascular events, chronic obstructive pulmonary disease, and placental villitis [5-10]. These observations suggest that these distinct tissue destruction processes share common final immune-mediated molecular mechanisms. We termed this phenomenon as the "Immunologic Constant of Rejection (ICR) [3]." The molecular constants shared among these different tissue destruction processes include the coordinated activation of the following pathways: I) STAT-1/IRF-1/T-bet/IFN-γ/IL-15 pathway; II) CXCR3 ligand chemokine pathway (CXCL9, -10, -11) III) CCR5 ligand chemokine pathway (CCL3, -4, -5) and IV) TIA-1 pathway/granzyme A/B/granulysin/perforin pathway [3,4].

Over the past decade gene expression microarrays have been employed to study allograft rejection in humans. The intrinsic heterogeneity among different investigators in terms of patient selection, microarray platforms, gene coverage, statistical analysis, sample collection and study design makes cross-comparison between different studies very challenging. Furthermore, since microarray profiling is a relatively new technology, it has continued to evolve in sophistication and has only recently become standardized [11,12]. For this reason we believe that despite the non-uniformity among studies, genes that are consistently reported across different studies and in different organs command attention. In this review we challenge the concept of the ICR by examining multiple studies to evaluate the presence of the "constants of rejection." We tested the ICR hypothesis by describing the most frequently reported immune pathways activated during acute allograft rejection in humans as reported by publications using microarray technologies. Biological explanations for relevant pathways are provided based on pertinent literature.

### Data Extraction Criteria

In this review we focused on high-throughput gene expression profiling studies which sought to characterize the molecular features of acute allograft rejection. Accordingly, we searched various combinations of the following MeSH terms/keywords in PubMed: "gene expression, " "acute, " "allograft, " "rejection, " and "microarray." Searches were performed independently by two investigators. Gene Expression Omnibus (GEO) and reference lists of original articles and review articles also served as additional search methods. Microarray studies providing original data and performed on human tissue biopsies during established acute allograft rejection were selected and evaluated [13-46]). Studies analyzing gene expression profiles of peripheral blood mononuclear cells and urine sediments during acute rejection will not be considered here, despite their potential utility as non-invasive diagnostic/predictive tools [47-51].

The compiled list of key genes in this review came from those reported as upregulated in the original publications, most of which were predominantly immune-related and are reported in Table 1. In total, 15 unique datasets met the search criteria, and comprise Tables 1 and 2. Of these datasets, four comparative analyses were among those selected for inclusion. Of note, all of the studies contained original data from *de novo *investigation.

**Table 1:** Key genes associated with acute allograft rejection according to human microarray studies

**Author: Tannapfel et al. 2001 **[14] Organ: Liver									
**IL3**	**MMP9**	**TGF-B1-3**	**TIMP1**	**TNF**	CLUSTERIN (CLU)	**IL10**			

**Author: Sreekumar et al. 2002 **[15] **Organ: Liver**									

**C1QB**	**C3**	LIPC	**GZMB**	HSPA1A	IGF1	**IL2**	(IRF9*)	**STAT1**	ACADM

**HLA-I**	**HLA-II**	PCK1	SELPLG	**TGFB1**	**TNF**	**TNFAIP3**	**TNFSF10**	UBB^3#^	UBA1

UBE2N	**ADAM17**	GYS2	CTPS						

**Inkinen et al. 2005 **[16] **Organ: Liver**									

**HLA-D**	**IL2RB**	**IL2RG**	**CASP1**	**CASP3**	**GZMA**	**GZMB**	SELL	ICAM3	ITGA4

SELE	VCAM1	**IFNG**	**IL1B**						

**Asaoka et al. 2009 **[28] **Organ: Liver**									

AKAP11	ALOX15	**CASP8**	CFLAR	FFAR3	**IFNAR1**	IGFBP3	**IL12RB1**	LTA	POU4F1

PPP1R8	PPP1R3A	PVRL1	TNK2						

**Author: Gimino et al. 2003 **[17]**Lande et al. 2006 **[30]**(MINNEAPOLIS Dataset)** **Organ: Lung**									

**C4B**	**CCR7**	**CD28**	**CD3E**	**CD84**	**CTLA4**	**CXCR3**	**GZMK**	**GZMA**	**IFNG**

IGKC	ITK	**KIR**	**PRF1**	**STAT4**	**IL2RA**	**IL2RB**	Zap70	LCK	

**Patil et al. 2008 (MINNEAPOLIS2 Dataset) **[18] **Organ: Lung**									

IFITM1	**CD8A**	MARCKS	**CCL3**	**GZMB**	ITM2A	**IL32**	**IL8**	**CCL4**	

**Author: Karason et al. 2006 **[19] **Organ: Heart**									

**C3**	**C4A**	**CXCL9**	**CXCL10**	**GBP1**	**HLA-C**	**HLA-F**	**HLA-J**	IGFBP4	NPPA

PSME2	**RARRES3**	**STAT1**							

**Author: Akalin et al. 2001 **[21] **Organ: Kidney**									

**Humig** **(CXCL9)**	**C3**	CD18 (ITGB2)	**ISGF-3** **(STAT1)**	MCL1	MIP-3β (CCL19)	NNMT	**RING4** **(TAP1)**	**TCRB^#^**	**IL2-SP^2#^** **(LCP1)**

**Author: Sarwal et al. 2003 (STANFORD Dataset) **[22] **Organ: Kidney**									

**HLA-A**	**HLA-B**	**HLA-C**	**HLA-E**	**HLA-DR**	**HLA-DQ**	**HLA-DMA**	**HLA-DRB4**	TGFBR2	TGFR1

**TCR**	DARC	**C4B**	**CXCL9**	**SCYA3** **(CCL3)**	**SCYA5** **(CCL5)**	**CCR5**	**SCYA2** **(CCL2)**	**CD20**	**PERFORIN**

CD53	**NFKB1**	**NK4 (IL32)**	**CX3CR1**	**GZMA**	**STK17B**	**IL6R**	**IL2RB**	**IL15RA**	**IL16**

**STAT1**	**CASP10**	**IFNGR1**	**IGHG3**	**IGKC**	**IGL**	**IGHM**	LENG4	CD59	VCAM1

**CXCL9**	**CXCL10**	**CCR5**							

**Author: Flechner et al. 2004 (CLEVELAND Dataset) **[23] **Organ: Kidney**									

**C1QB**	**CCL5**	**CD14**	CD163	**TRB@**	CD16	CD2	CD27	**CD3D**	CD48

CD53	**CD64 (FCGR1A)**	**CD8**	CDW52	**CXCR4**	**GZMA**	**HLA-F**	**IFI30**	**IL10RA**	**IL10RB**

**IL4R**	ISG20	PKR (PRKRA)	RAGE4 (RAGE)	TNFRSF1b					

**Reeve et al. 2009 (EDMONTON Dataset) **[24] **Organ: Kidney**									

APOBEC3G	**CCL4**	**CCL5**	**CD8A**	CRTAM	**CXCL9**	**CXCL10**	**CXCL11**	FAM26F	**GBP1**

**GBP2**	GBP4	GBP5	**GZMA**	**GZMB**	INDO	**LCP2**	LILRB1	NLRC5	**PSMB9**

**Author: Morgun et al. 2006 (SAN PAULO Dataset) **[25] **Organ: Heart + Lung + Kidney** **(SAN PAULO+ MINNEAPOLIS+STANFORD+CLEVELAND Datasets)**									

ABCA7	CD14	DAP10	**HLA-A**	HSD17B7	ISG20	LEF1	NT5C2	RU2	TRAF2

**ADAM15**	CD2	F2R	**HLA-B**	HSPC043	ISGF3G (IRF9)	LIAS	PAK4	SELPLG	TRB@

**ADAM8**	CD3Z	FCER1G	**HLA-DMA**	HSPC129	ITGB2	LILRB4	PCDHGA8	SLC14A2	**UBD**

B2M	CD53	FKBP14	**HLA-DMB**	IFI30	KCNJ5	LOC90586	PSCD4	SMG1	UBE1L

BAX	CD7	FLJ10244	**HLA-DQB1**	IGHG3	KCNK6	LOC92033	PSMB9	SORL1	UBE2B

BTN3A3	CD74	FLJ11106	**HLA-DRA**	IGKC	KIAA0924	LTB	PSME1	**STAT1**	UBE2L6

**C1QA**	CG012	FLJ11151	**HLA-DRB1**	IGLC2	KIAA1030	LTB4R	RAB7L1	SULT1A3	UCP2

**C4A**	CHD3	FLJ11467	**HLA-DRB3**	IGLC6	KIAA1170	MAFF	RAC2	TAP1	**WSX1 (IL27RA) **

**CASP4**	CORO1A	FY	**HLA-DRB5**	IGLJ3	KIAA1257	MSH3	**RARRES3**	TAPBP	ZAP70

CCL18	CTSS	GMFG	**HLA-E**	**IL2RB**	KIAA1348	NKG7	RASGRP2	TCBRV (IL23A)	ZNRD1

**CCL5**	**CX3CL1**	**GZMK**	**HLA-F**	**IRF3**	LAT	NM23-H6	RBL1	TNFAIP3	TNFSF13B

**CXCL9**	D21S2056E	HA-1	**HLA-G**	**IRF5**	LCK	NPHP1	RIMS1		

**Reference: Saint-Mezard et al. 2008 (PARIS Dataset) **[26] **Organ: Kidney** **(PARIS+STANFORD+CLEVELAND+NON HUMAN PRIMATES Datasets)**									

ARHGDIB	ARPC2	**CASP1**	**CASP4**	**CCL5**	CD163	**CD44**	CD48	CD52	CD53

**CD8A**	CSPG2	**CXCL10**	**CXCL9**	FCER1G	FER1L3	**GBP1**	**GBP2**	GMFG	**GZMA**

HCK	HCLS1	**HLA-B**	**HLA-C**	**HLA-DMA**	HLA-DMB	**HLA-DPA1**	**HLA-DQB1**	**HLA-DQB2**	**HLA-DRA**

**HLA-DRB3**	**HLA-E**	**HLA-F**	**HLA-G**	**IFI30**	IFITM1	IGHM	**IL10RA**	ISG20	ITGB2

LAPTM5	**LCK**	LCP1	**LCP2**	LTF	LYZ	**MMP7**	NMI	PLEK	PLSCR1

PRG1	PRKCB1	PSMB10	**PSMB8**	**PSMB9**	RAC2	RUNX3	SERPING1	SLA	**STAT1**

**TAP1**	TCIRG1	**TIMP1**	TNC	TNFRSF7	**UBD**	UBE2L6	WARS	WFDC2	T3JAM (TRAF3IP3)

**Rodder et al. 2010 (TENON/INSELSPITAL Dataset) **[29] **Organ: Kidney** **Meta-analysis focused on metzincins and related genes** **(TENON/INSELSPITAL+EDMONTON+STANFORD+ CLEVELAND Datasets)**									

**TIMP1**	**MMP7**	ADAMTS18	ADAMTS6	**ADAMTS17**	**ADAMTS8**	ADAMTSL4	ADAM18	TLL2	**ADAM17**

PLG	LAMA4	EMILIN2							

**Chen et al. 2010 (STANFORD2 dataset) **[27] **Organs: Kidney, Heart** **(STANFORD2+CLEVELAND+SAN PAULO Datasets)**									

**CXCL9**	**CXCL11**	**CXCR4**	**STAT1**	**CCL4**	C6orf32	MARCKS	IGSF6	CD2	TRPM1

**IL10RA**	**RARRES3**	NR4A2	PTPRC	LEF1	TAP1	CTSS	ISG20	CCL8	BASP1

SLC2A3	LCP2	**HLA-DMA**	BIRC5	**HLA-DMB**	**CASP4**	SELL	HLA-F	**CD44**	**HLA-DQB1**

PIK3CD	**PECAM1**	MDK	MELK	CDKN3	CPD	SH2D2A	CCNB2	HLA-DRA	B2M

DIAPH1	USP34	SCAND2	RUNX1	S100A4					

Genes underlining highly redundant themes among studies are in bold.

This table reports upregulated genes associated with acute allograft rejection detected by microarrays technology analyzing human graft samples (bronchoalveolar lavage for lung samples, tissue biopsies for the other samples). Additional detail regarding the data extraction is provided in Additional file 1.

*Synonymous gene symbols, according to NCBI Gene, are provided in brackets.

^#^The original name reported in the publication was: TCR Active β-chain related gene (M12886: unmapped). ^2# ^The original name reported in the publication was: IL-2-stimulated phosphoprotein. ^3#^The original name reported in the publication was: ubiquitin.

**Table 2:** Characteristics of microarray studies evaluating gene expression profile in acute allograft rejection biopsies in humans.

Author (dataset) * Year Organ (samples)	Array^†^	Aim/Design^‡^
Tannapfel et al. [14] 2001 Liver (biopsies)	Atlas human cDNA ~ 600 genes	**Aim**. To investigate the expression of multiple inflammatory and apoptosis related genes in acute allograft rejection. ** Design**. (Adults) 62 patients, 97 biopsies: acute allograft rejection (n = 32), HCV reinfection (n = 18), CMV infection (n = 5), acute rejection and HCV infection (n = 3), stable graft function (n = 30) and after treatment of acute rejection (n = 9). ** Statistics**. Not available.

Sreekumar et al. [15] 2002 Liver (biopsies)	Affymetrix HU 6800 ~ 6, 400 genes	**Aim**. To study intragraft gene expression patterns in acute cellular rejection and during recurrence of HCV in HCV infected recipients. **Methods**. (Adults) 8 patients and biopsies: HCV infection and acute cellular rejection (n = 4), HCV infection without acute cellular rejection (n = 4). **Statistics**. T-tests and fold change threshold.

Inkinen et al. [16] 2005 Liver (biopsies)	Turku Centre of Biotechnology human immunochip ~ 4, 600 genes	**Aim**. To determine and compare gene signature of CMV infection and acute rejection. **Methods**. (Adults) 7 patients and biopsies: CMV infection (n = 4), patients with acute rejection (n = 3). Gene expression of CMV and acute rejection samples were compared to that of liver graft after reperfusion. **Statistics**. Not available.

Asaoka et al. [28] 2009 Liver (biopsies)	AceGene Human chip ~ 30, 000 genes	**Aim**. To identify genes characteristic of acute cellular rejection in patients with recurrent HCV infections. **Methods**. (Adults) 21 HCV positive patients, 22 biopsies: acute cellular rejection (n = 9), without acute cellular rejection (n = 13). The expression of some transcripts (CASP8 and BMP2) was validated through qRT-PCR in this data set and also in a validation set: 32 biopsies from 25 HCV positive patients. ** Statistics**. Class discovery: unsupervised clustering analysis. Class comparison: Mann Whitney U test, supervised cluster analysis. Biological explanation: networks were built by Ingenuity Pathway Analysis (IPA).

Gimino et al. [17] (Minneapolis Dataset) 2003 Lung (BAL)	Affymetrix Human Genome U133A ~ 18, 000 genes	**Aim**. To determine markers of acute rejection in lung recipients. ** Methods**. (Adults) 26 patients, 34 samples: acute rejection (n = 27), without diagnosis of rejection (n = 7). **Statistics**. Class comparison: significance analysis of microarray. Class description: supervised clustering analysis.

Patil et al. [18] (Minneapolis2 Dataset) 2008 Lung (BAL)	Affymetrix Human Genome U133A ~ 18, 000 genes	**Aim**. to improve acute rejection diagnostics by identifying genes whose expression best classifies acute rejection versus no rejection **Methods**. (Adults) 32 patients, 32 samples: acute rejection (n = 14), without diagnosis of rejection (n = 18). Expression of some transcript was also assessed through qRT-PCR. ** Statistics**. Class comparison: Significance analysis of microarrays. Class prediction: prediction analysis of microarrays, method of nearest shrunken centroids with 10 fold cross validation. Biological explanation: Gene Ontology and GoHyperG.

Karason et al. [19] 2006 Heart (biopsies)	Affymetrix Human Genome U133A ~ 18, 000 genes	**Aim**. To utilize microarray analysis to search for potential biomarkers of cardiac allograft rejection. ** Design**. (Adults). 20 patients, 14 patients experienced acute rejection episodes. 3 patients with acute rejection and biopsy available at three different time-points (before: normal histology, during: biopsy with acute rejection episode, after: biopsy with normal histology after the rejection episode) were profiled. qRT-PCR was performed for selected genes (CXCL9, CXCL10, NNPA). Serum levels of CXCL9 and -10 in 10 patients at three time points were also determined. ** Statistics**. Gene clustering according to time-point analysis: self organizing map (SOM) algorithm. Biological explanation: Gene Ontology (GO) and Netaffx.

Akalin et al. [21] 2001 Kidney (biopsies)	Affymetrix HU 6800 ~ 6, 400 genes	**Aim**. To analyze gene expression profile using microarrays in acute allograft rejection. ** Design**. (Adults) 10 biopsies: histological evidence of acute cellular rejection (n = 7), without evidence of rejection (n = 3). ** Statistics**. Each acute rejection sample was compared with each control sample. Genes with a > fourfold increase in the majority of the samples were selected.

Sarwal et al. [22] (Stanford Dataset) 2003 Kidney (biopsies)	Lymphochip > 12, 000 genes	**Aim**. To investigate the possibility that variations in gene-expression patterns in allograft-biopsy samples from patients with acute rejection and related disorders could identify molecularly distinct subtypes of acute rejection to possibly explain differences in clinical behavior. ** Design**. (Pediatric patients) 50 patients. 67 biopsies: biopsies during acute or chronic allograft dysfunctions (n = 52) and at the time of the engraftment or when graft function was stable (n = 15). The possibility of different sampling of the medullary and the cortical regions was also addressed. ** Statistics**. Class discovery: unsupervised clustering analysis. Class comparison: significance analysis of microarray. Survival analysis: Kaplan-Meyer/Cox log-rank method. Biological explanation: enrichment of specific functional groups through evaluation of hypergeometric distribution. The exclusion of data from genes whose expression was correlated with the depth of biopsy did not change the cluster analysis.

Flechner et al. [23] (Cleveland Dataset) 2004 Kidney (biopsies and PBLs)	Affymetrix HG-U95Av2 ~ 10, 000 genes	**Aim**. To determine gene expression profiling in transplant patients including: normal donor kidneys, well functioning transplants without rejection, kidneys undergoing acute rejection, and transplants with renal dysfunction without rejection. ** Design**. (Adults) 23 graft recipients and 9 donors. Acute rejection biopsies (n = 7), renal dysfunction without rejection on biopsies (n = 6), biopsies carried out more than one year post transplant in patient with good transplant function and normal histology (n = 10), biopsies from living donor controls (n = 9). PBLs were also collected and profiled. Expression of some transcript was also assessed through qRT-PCR. ** Statistics**. Class discovery: unsupervised clustering analysis. Class comparison: significance analysis of microarray filtered with limit fold model and MAS 5.0 present/absent calls. Class-prediction: leave-one-out method. Biological explanation: analysis of functional classes of the differentially expressed genes.

Reeve et al. [24] (Edmonton Dataset) 2009 Kidney (biopsies)	Affymetrix Human Genome U133 Plus 2.0 > 38, 000 genes	**Aim**. To define a classifier to distinguish rejection vs non rejection using predictive analysis for microarrays. **Design**. (Adults) 143 patients, 186 biopsies: acute rejection samples (acute cellular rejection, antibody mediated rejection or mixed) (n = 51), non-rejection samples (n = 135). **Statistics**. Class comparisons: Bayesian t-test and false discovery rate. Class prediction: prediction analysis of microarrays. Biological explanation: analysis of functional classes of the differentially expressed genes according to KEGG pathways and to authors' defined pathogenesis-based transcripts.

Morgun et al. [25] (San Paulo Dataset) 2006 Heart (biopsies)	Qiagen/Operon Array ~ 14, 000 genes	**Aim**. To analyze gene expression differences between rejection, non rejection and Trypanosoma cruzi infection. ** Design**. (Adults) 40 patients, 76 biopsies (rejection, no rejection and Trypanosoma cruzi infection recurrence). Expression of some transcripts was also assessed through qRT-PCR. **Statistics**. Class comparison: random variance t-test filtered with univariate/multivariate tests for false discovery rates; supervised clustering analysis. Class prediction: 6 different multivariate models models (compound covariate predictor, diagonal linear discriminant analysis, 1- and 3-nearest neighbor predictor, nearest centroid predictor, support vector machine) and leave-one-out cross validation. The authors validated the predictor-set in independent datasets of biopsies (collected on different continents and analyzed with different chip batches). The authors also tested the predictor set by analyzing the data from data from Cleveland (Kidney) Stanford (Kidney) and Minneapolis (Lung) datasets. Biological explanation: Database Annotation, Visualization and Integrated Discovery (DAVID)/Gene Ontology and KEGG Pathways.

Saint-Mezard et al. [26] (Paris Dataset) 2008 Kidney (biopsies)	Affymetrix Human Genome U133 Plus 2.0 > 38, 000 genes	**Aim**. To identify a robust and reliable molecular signature for acute rejection in humans. ** Design**. (Adults) 45 patients, 47 biopsies: acute rejection (n = 8), acute rejection and chronic allograft nephropathy (n = 8), borderline (n = 3), non rejection (n = 7), and chronic allograft nephropathy (n = 22). Normal kidney tissue was obtained from histopathologically unaffected areas of the cortex of native nephrectomies performed for renal carcinoma was used as control. **Statistics**. Genes differentially expressed (Paris Dataset) were intersected with those from with 2 public human datasets: 1) Stanford Dataset and 2) Cleveland Dataset and with one Non Human Primate (NHP) model of acute renal allograft. However, the authors used biopsy microarray data from Edmonton Dataset as in independent confirmation set. Score from the identified classifier was correlated with the histopathological Banff score. Expression of some transcripts was also assessed through qRT-PCR. Class comparison: ANOVA with or without false discovery rate and additional cutoff based on twofold change. Class discovery: Principal component analysis, supervised clustering analysis (using the genes differentially expressed in all four datasets); Class prediction: leave-one-out cross-validation and 10-fold cross-validation. Biological explanation: Gene regulatory networks were generated using MetaCore.

Rodder et al. [29] (Tenon/Inelspital Dataset) 2011 Kidney (biopsies)	Affymetrix Human Genome U133 Plus 2.0 > 38, 000 genes	**Aim**. To identify the expression of metzincins and related genes in allograft rejection biopsies. ** Design**. (Adults) 41 biopsies: normal histology (n = 20), borderline changes (n = 4), acute rejection (n = 10) and acute rejection and interstitial fibrosis/tubular atrophy (n = 7). Expression of some transcripts was also assessed through qRT-PCR. ** Statistics**. Class prediction: ANOVA and shrinking centroids methods were used for variable selection and a variety of classification methods were tested. Leave-one-out method was performed as internal cross-validation. Classifier performance was estimated as correct rate after 1-level cross validation. The model was validated in Edmonton, Cleveland and Stanford datasets. Gene set scores from biopsies were also determined and correlated with Banff scores.

Chen et al. [27] (Stanford2 Dataset) 2011 Kidney (biopsies)	Affymetrix Human Genome U133 Plus 2.0 > 38, 000 genes	**Aim**. To identify biomarkers across similar conditions through integration of related datasets. **Methods**. (Pediatric patients) 36 patients and biopsies: acute rejection biopsies (n = 18), stable function biopsies (n = 18). ** Statistics**. Class comparison: significance analysis of microarrays and fold change filter. The upregulated genes during acute rejection were intersected with genes upregulated during acute rejection in two other datasets (Cleveland and San Paulo).

Notes

*For the Minneapolis Dataset only the publication by Gimino et al. is described;

^†^Microarray chips details: Atlas human cDNA microarrays ~ 588 gene analyzed; Affymetrix GeneChip HU6800 Array containing > 7, 000 oligonucleotide probe sets representing ~ 6, 400 human genes (Affymetrix, Santa Clara, CA);

Affymetrix Human Genome U133A Array containing > 22, 000 oligonucleotide probe sets representing > 18, 000 transcripts (~ 14, 500 human genes) (Affymetrix, Santa Clara, CA); Lymphochip: in-house microarrays containing > 28, 000 cDNA probes representing > 12, 000 genes (Stanford University); Affymetrix GeneChip HG-U95Av2 Array containing ~ 12, 000 oligonucleotide probes representing ~ 10, 000 human genes; Affymetrix Human Genome U133A Array containing > 22, 000 oligonucleotide probe sets representing > 18, 000 transcripts (~ 14, 500 human genes) (Affymetrix, Santa Clara, CA); Affymetrix Human Genome U133 Plus 2.0 Array containing > 54, 000 oligonucleotide probe sets representing > 47, 000 transcripts (~ 38, 500 human genes) (Affymetrix, Santa Clara, CA); Qiagen/Operon array: in-house oligonucleotide array platform designed by Qiagen/Operon (Alameda, CA) and printed at NIAID Microarray facility, representing ~ 14, 000 human genes;

^‡^Study aim/design is referred to gene expression experiments;

Abbreviations: BOS: Bronchiolitis obliterans syndrome; PBLs: Peripheral blood lymphocyte; CMV: cytomegalovirus; HCV: hepatitis C virus; qRT-PCR: quantitative real time polymerase chain reaction;

The Ingenuity Systems Pathway Analysis (IPA) http://www.ingenuity.com and MetaCore http://www.genego.com were used to illustrate the relationships among the compiled list of key genes. Additional detail regarding the data extraction is provided in Additional File 1.

### Overview of microarray studies

Considering the heterogeneity among the selected studies in terms of platform used, purpose, design, and interpretation (see also Table 2), a quantitative approach was not feasible, making this review qualitative in nature. The diversity of the clinical setting (pediatric or adult patients; heart, lung, liver or kidney transplants) also added complexity to this analysis. The purposes of the original studies included here ranged between class discovery, class comparison, and/or class prediction. Different methods (summarized in Table 2) were used by different investigators to provide a list of genes modulated during acute allograft rejection. Not surprisingly, little overlap exists among studies with respect to specific 'genes' described as upregulated during acute rejection, yet, we found a striking consistency in terms of pathway overrepresentation suggesting that each individual study identified different pieces of the same puzzle.

It should be noted that these studies lacked the use of micro/macro-dissection which prohibited identification of the cellular source of the transcripts differentially expressed during acute rejection. It is logical to think that analysis of RNA from the whole tissue samples could influence gene expression patterns. With this in mind, Sarwal et al. [22], investigated if the molecular changes observed during allograft rejection could have been related to the differential sampling of cortical and medullary kidney sections. For this analysis the authors excluded the genes whose expression was shown to be correlated with the depth of biopsy in a previous investigation. The introduction of this filter did not significantly change the results. In another study involving kidney recipients, Rodder et al. [29] performed qRT-PCR on isolated glomeruli, and on proximal and distal tubules. Although qRT-PCR of targets genes (metzincins and related genes) revealed some differences between glomeruli and tubules, it confirmed, overall, the differences between acute rejection and normal samples detected by microarray analysis.

### The Immunologic Constant of Rejection pathways in acute allograft rejection

**Figure 1:**
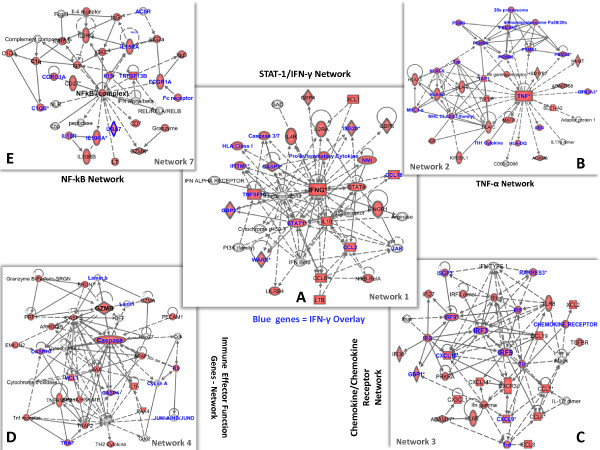
**First 5 Immune networks according to Ingenuity Pathways Analysis (IPA), representing schematic relationships among key genes upregulated in acute allograft rejection (Network number 1 (A), 2 (B), 3 (C), 4 (D) and 7(E), generated by IPA)**. The gene list uploaded represents the *key gene list *(Table 1). Red: The genes and gene complexes from the *key gene list *are represented in red background (no color fill is used for the genes that are part of the network but not part of the *key gene list*). Blue: IFN-γ stimulated genes (designated IFN-γ stimulated genes identified as those upregulated in peripheral monocytes after IFN-γ stimulation). **A**. the first network is centered around IFN-γ; **B**. the second network is centered around TNF- α; **C**. the third network focuses on Interferon Regulatory Factors (IRFs) and chemokine/chemokine receptor interaction (i.e., CCR5/CCR5 ligands and CXCR3/CXCR3 ligands); **D**. the fourth network focuses on Immune Effector Function (IEF) genes (i.e., around granzyme B, perforin, caspases); **E**. the fifth network is centered around the NF-kB complex. Bold lines indicate direct interaction. Dotted lines indicate indirect interaction.

After reviewing the literature, we found that pathways involved in the ICR hypothesis are frequently activated during acute allograft rejection across studies conducted by different investigators in different organs (see Table 1). Figure 1 provides a visualization of the relationships among the key genes described. Here, we attempt to illustrate the hypothetical role of these pathways during acute allograft rejection.

#### IFN-γ pathway

IFN-γ is a pleiotropic cytokine that plays a role in the modulation of many aspects of the immune response. Studies conducted involving IFN-γ -/- mice suggest that this cytokine, in addition to its pro-inflammatory functions, might be important for graft acceptance, preventing early graft necrosis, and maintaining microvascular viability [52,53]. However, the molecular mechanisms through which IFN-γ exerts its anti-inflammatory action during the early phases of the engraftment are unclear. On the other hand, this interferon is considered a central cytokine in sustaining inflammation during allograft rejection both in humans and in murine models.

IFN-γ is predominantly produced by NK cells as part of the innate immune response, and by CD4 T helper 1 cells (Th1) and CD8 cytotoxic T cells (CTLs) as a part of the adaptive immune response once antigen-specific immunity develops [54,55]. Its overexpression has also been observed in acute allograft rejection in several human studies where RT-PCR was applied [17,56-58]. Microarray studies have not only enabled the detection of the expression of the IFN-γ gene itself, but also the detection of its downstream effects (IFN-γ stimulated genes) [15,17,21-24,59]. Figure 1A represents the first network generated by IPA after analysis of the compiled list of key immune-related genes (Table 1), with IFN-γ serving as the hub of this important network. Detection of IFN-γ stimulated genes alone is not sufficient to discriminate its effect from the effects of other cytokines, such as IFN-α, which can also activate many of the IFN-γ stimulated genes [60,61]. However, the prevalence of IFN-γ versus IFN-α transcripts in addition to the activation of pathways that specifically enhance the INF-γ loop (e.g. TNF-α, CCR5, and CXCR3) implicate IFN-γ as a driver gene involved in sustaining acute allograft rejection [22,24,26,31,59,62]. Although some functions have been described for individual IFN-γ stimulated genes, the overall orchestration is not completely understood. A partial description of the relationship among IFN-γ stimulated genes detected in microarray studies is illustrated in Figure 1A.

Primarily through interferon regulatory factor 1 (IRF-1), IFN-γ upregulates both MHC class I and II, by increasing the expression of antigen peptide transporters TAP1-2, or class II transactivator CIITA, for example [54,63,64]. Indeed, IFN-γ promotes the differentiation of naïve CD4 T cells into Th1 cells which are, among the lineage of CD4 T cells (Th1, Th2, Th17 and T Reg), the only ones that produce a consistent amount of IFN-γ [55,65]. IFN-γ in turn, and usually in synergy with tumor necrosis factor-α (TNF-α), induces the expression of CXCR3 ligands [54,66,67] and CCR5 ligands [68].

#### CXCR3 and CCR5 ligand pathways

CXCR3 ligands (CXCL9, -10, -11) and CCR5 ligands (CCL3, -4, -5) are the chemokines most frequently upregulated during acute allograft rejection as described by human microarray studies [18,21-27] and RT-PCR (See Table 1). The upregulation of the related receptors, CXCR3 and CCR5, has also been frequently described, though not as much specifically within microarray studies [17,22,24,69-72]. Interestingly, high urinary CXCR3 ligand protein levels were detected in clinical trials in patients experiencing acute kidney rejection [48-51]. More recently, Chen et al. [27] described CXCL9 as a biomarker of acute rejection in a cross-organ microarray study evaluating pediatric renal, adult renal, and adult cardiac transplant patients. Additionally, CXCL9 proteins were also found to be elevated in serum. Indeed, the CCR5Δ32 polymorphism that encodes for a nonfunctional CCR5 receptor, conferred a greatly reduced risk for the development of acute rejection in kidney [73] and in liver transplantation [74].

The driving role of these two pathways in allograft rejection was suggested by *in vivo *murine models one decade ago [75-78]. The lack of host CCR5 was associated with a three-fold increase in allograft survival rate, but the targeting of any of the three main ligands using knockout mice or monoclonal antibodies had no effect on allograft survival [79]. Similarly, lack of CXCR3 led to graft acceptance indefinitely [75] and in addition, a significant survival benefit has been observed in CXCL10 -/- recipients [76]. This supposed non-redundant effect of CXCR3 that has been assumed correct for almost ten years, is currently object of debate [80]. Recently, in fact, two independent studies reported that disruption or blockade of recipient CXCR3 had relatively little effect on rejection [81-83]. These observations, as well as the abundance of CXCR3 ligands present during acute allograft rejection, raise questions about the functional importance of CXCR3 during rejection and the possibility of alternative targets of CXCR3 ligands [80].

Upon antigen stimulation, the co-expression of CXCR3 and CCR5 is a marker of Th1 cell polarization, whereas CCR3, CCR4, CCR8, and CRTh2 are expressed by Th2 cells [65]. The genes and gene pathways frequently overexpressed during acute allograft rejection are consistent with the predominance of Th1 cell polarization. Among CXCR3 and CCR5 ligands, CXCL9, -10 and CCL4, -5 were the most frequently reported chemokines associated with acute rejection in microarray studies [19,21-24,31] (see Table 1).

CCR5 and CXCR3 ligands can be secreted differentially by dendritic cells, activated macrophages and T cells, endothelial cells, and NK cells [69,70,84-87]. However, studies that define the cell-specific production of chemokines in allograft rejection in humans are scant. Consequently, evidence-based descriptions of cell-specific chemokine-mediated recruitment have not been well-defined in humans either.

Hoffmann et al. [88] described that a significant proportion of both CD4+ and CD8+ T-cells detected in human renal biopsies during rejection express CXCR3. It has also been speculated that CXCR3 may act as a decoy receptor by binding CCL11, preventing the recruitment of granulocytes via the CCL11/CCR3 binding interaction [80,89]. This might explain why granulocytes are not classically found in acute cellular rejection. Furthermore, peripheral blood monocytes that are lower in CCR5 expression could be recruited through CCR1 that also binds CCL-3, -4, -5. In addition to Th1 and CTL cells, NK cells could also possibly be recruited through this pathway since they are all known to express CXCR3 and CCR5 receptors [85]. However, NK cells are rarely present in allograft infiltrates and are especially rare in T-cell mediated rejection [39,90].

Recruitment and activation of CCR5 and CXCR3 ligands can lead to increased production of IFN-γ, with a resultant amplification of the inflammatory stimuli and further release of chemoattractant molecules. Thus, in a concerted fashion, these molecules orchestrate the switch from innate to adaptive immunity, meanwhile sustaining and strengthening the innate cytotoxic mechanisms with a persistent "NK-like" response.

Finally, even though up to 25% of circulating B cells express CXCR3 [90-92], and can also produce CXCR3 ligands [87,93], the recruitment of B cells through this mechanism during acute allograft rejection has not yet been defined in humans.

This complex cascade of cytokines and the coordinate activation of specific pathways so far described, leads to the activation of IEF genes (perforin, granzymes A/B, Fas/Fas ligand, and caspases) during the process of tissue destruction.

#### IEF genes

The release of granzymes, perforins, and granulysin and the interaction between the Fas/Fas ligand and caspase activation represent the major effector mechanisms of cell-mediated immunity [94]. These IEF transcripts have been consistently described as being associated with acute allograft rejection using transcriptome analyses [17,22,23,26].

By profiling PBMCs, Hidalgo et al. [40] found that cytotoxic molecular transcripts (i.e. granzyme B, Fas ligand, perforin) are commonly overexpressed in CTL CD4+ cells, CTL CD8+ cells, and NK cells. These observations highlight the existence of a common molecular cytotoxic "NK-like" effector mechanism that is shared among the different arms of the immune system, the classically distinct innate and adaptive immune arms. Taking this one step further, Mueller et al. [31] found that gene expression patterns of T-cell mediated rejection are surprisingly similar to the expression patterns found in antibody-mediated rejection. In particular, interferon-γ affected transcripts and IEF genes such as perforin, granzyme B, and Fas ligand were overexpressed in both of them. This observation suggests that effector T cells and antibodies lead to the activation of a common final pathway in tissue destruction and supports the proposed theory of the immunologic constant of rejection [3].

### NK cell, B cell, and T-reg signatures

#### NK signature

NK cells in murine skin and rat liver allografts are the immune cells responsible for early chemokine production of CCR5 and CXCR3 ligands (i.e., CCL3, CCL4, CXCL10) which are important in initiating and sustaining acute allograft rejection [95,96]. Nevertheless, NK cells do not seem to be sufficient to reject solid organs directly since mice that have intact NK cell function but absent adaptive immunity (RAG^-/- ^or SCID) are able to accept skin and cardiac graft transplants indefinitely [97-99]. However, the inability to reject the graft does not prove that innate cells (in this case NK cells) are unable to mediate tissue destruction. A possible explanation could be that, in these models, the lack of stimuli derived from a reciprocal feedback between innate and adaptive cells, does not allow triggering or sustaining a strong enough "NK-like" cytotoxic effector function.

Recently it has been observed that nude mice treated with oncolytic viruses can reject tumor xenografts [100]. This rejection was associated with the activation of ISGs (both IFN-γ and IFN-α stimulated genes), upregulation of CXCR3 and CCR5 ligands, and activation of IEF genes (granzyme B, caspase 8). Since these mice lack T cells and secondarily lack B cell responses, this immune-mediated tissue destruction is thought to be induced by innate immune effectors such as NK cells and activated macrophages. This study suggests that, at least in this model, innate immunity can be an independent effector of tissue-specific destruction not requiring adaptive immunity. It is possible that the oncolytic virus used in this model primes the innate immune system in a manner that bypasses the need for the adaptive immune system interaction.

In humans, however, studies analyzing the individual contribution of innate immune cells in mediating the final step of the alloresponse are lacking. Although NK cells are present, they are only a minor component of allograft tissue infiltrates in acute rejection [98,101,102]. For this reason they are traditionally thought to exert only a marginal role. Therefore, the study conducted by Hidalgo et al. [39] was remarkably revealing. The investigators compared the gene expression profiles of antibody-mediated rejection in humans by analyzing the gene expression profiles in biopsies from patients with donor specific antibody. In these antibody-mediated rejection samples there was a strong expression of IFN-γ associated transcripts and NK cells. Immunohistochemical staining displayed more NK cells and macrophages in antibody-mediated rejection than in T-cell-mediated rejection. These findings suggest that the frequent observation of IEF gene upregulation not only during T cell mediated rejection but also during antibody mediated rejection could reflect the activation of common "NK-like" effector functions. Recently, tolerant patients have been found to have an expansion of NK cells and overexpression of NK transcripts in peripheral blood. These findings helped generate new hypotheses on the role of NK cells in balancing tolerance and mediating rejection [103].

#### B cell signature

The precise role that B cells play in acute allograft rejection is still being discovered. Recent high-throughput studies have exposed the multi-faceted role of B cells within allografts. With some evidence to suggest a significant role for B cells in mediating rejection, other evidence also suggests a role for B cells in tolerance. The B cell is an enigma and the details of its functions must be further elucidated.

In a breakthrough paper in 2003, Sarwal et al. [22] found an enrichment of B cell transcripts in pediatric renal biopsies experiencing acute rejection. This unexpected B-cell signature was also confirmed by immunohistochemistry. Although ectopic germinal center formation with B-cell activation and plasma cell activation can occur locally in chronically inflamed tissue [104,105], the *in situ *presence of B cells was not historically reported in acute allograft rejection [22,106,107]. This CD20+ B cell infiltration was not associated with intra-graft C4d deposition (required by Banff criteria for diagnosis of acute antibody-mediated rejection) [108] or with the detection of donor-specific antibodies, indicating that it was not necessarily related to the presence of humoral response. The presence of such CD20+ dense clusters in a significant proportion of samples from patients diagnosed with acute allograft rejection would, thereafter, be confirmed by several independent studies [109-116], with incidence varying from 15% to 60% [38,113,116]. However, the correlation with poor outcome suggested by Sarwal et al. [22] was confirmed by some studies [112,114,115,117] but not by others [38,109-111,113] and seemed more likely associated with late inflammation in allograft rejection [38]. B cell transcripts have also been detected to be upregulated during acute rejection in lung and heart transplants (Table 1) [17,25].

The subsequent lineage analysis revealed that CD27+ and chronically activated CD79+, CD20+ B cells expressed HLA antigens and were surrounded by CD4+ T cells. This suggests a putative role for these cells in antigen presentation, driving a T-cell dependent cellular rejection [118]. Another cluster of B cells was represented by CD138+ mature plasma cells [118]. Recently, studies conducted in heart transplantation models showed that a deficiency in B cell mediated antigen presentation leads to lack of CD4 T cell activation and alloantibody production [119]. Other *in vivo *observations pointed to the possible pivotal role of B cells in the context of pathogen- [120,121] or autoimmune- [122] induced T cell response. Interestingly, B cell infiltrates have been associated with favorable outcome in early breast cancer patients [123]. However, in addition to functioning as antigen presenting cells, B cells may promote T cell mediated rejection by producing chemoattractant molecules such as CXCR3 ligands (CXCL9, -10, -11) [87].

Despite the elegant rationale behind the use of an anti-CD20 monoclonal antibody (rituximab) in transplantation, this treatment showed only partial efficacy when tested in randomized trials [124-126]. Although rituximab depletes CD20+ CD27- naïve and CD20+ CD27+[127] memory cells, it is not active on plasma cells that are CD20- and are thought to be implicated in the pathophysiology of acute antibody-mediated rejection. Additionally, two high-throughput studies evaluating several parameters in peripheral blood [103,128] and urine [128] of patients with drug-free spontaneous renal allograft tolerance found an expansion of B-cells in peripheral blood, confirming a previous report [129]. The particular phenotype of these B cells seems to be represented by an expression of memory activated B-cells and increased expression of inhibitory molecules [130]. These observations could explain the increased rate of rejection reported in rituximab-treated patients in a recent randomized controlled trial that was forced to stop prematurely [126]. With B-cells implicated in both rejection and in tolerance, their precise functions remain puzzling.

#### T-reg signature

The recent detection of the association between the transcription factor forkhead box 3 (FOXP3) transcripts and acute rejection deserves comment. The recruitment of CD25+, FOXP3+ T regulatory cells (T-regs) is a well-defined mechanism for controlling autoimmunity in humans and animal models. It is known that humans carrying X-linked FOXP3 mutations manifest an autoimmune syndrome consisting of immune dysregulation, polyendocrinopathy and enteropathy, termed IPEX syndrome. Additionally, FOXP3 knockout mice manifest severe autoimmune diseases as well [131,132]. However, the presence of FOXP3+ cells and/or the expression of FOXP3 are not always associated with a decreased immune response and their biological significance remains unclear. Interestingly, the pre-treatment presence of FOXP3+ T cells was associated with favorable outcome in colon cancer patients undergoing chemotherapy or immunochemotherapy [133,134]. In kidney transplantation, however, higher FOXP3 transcripts in cells obtained from urine samples was associated with acute rejection [135]. Additionally, in another study, FOXP3 expression was found to be higher in antibody-mediated and T-cell mediated acute rejection samples than it was in the non-rejection samples [136]. Since FOXP3 mRNA directly correlated with post-transplantation time the authors speculated that FOXP3 positive cells possessed the key to control the potential for autoimmunity in these sites rather than representing a cognate immune-response. Nevertheless, it is presently unclear if FOXP3 (acting as a transcription factor) can modulate the immune-response *per se *through unknown independent pathways.

### TNF*-α*, Complement and IL-10: the link between the innate and adaptive immunity

#### TNF-α

The upregulation of the TNF-α pathway is another signature often associated with acute allograft rejection (Table 1, Figure 1B). Many of the genes expressed during allograft rejection are associated with innate immunity: TNF-α, ubiquitin, C3, Heat shock protein 70 (HSPA1A, which is the endogenous ligand of Toll-like receptor (TLR)-4) [137,138] and IRF-9 (a protein that interacts with STAT-1 and STAT-2 to form ISGF3, a transcription factor for IFN-α) [139,140].

The presence of TNF-α is not indicative of acute inflammation, and it is typically also present in chronic inflammation [3,141,142]. Although the transformation from an indolent process to an acute one is unknown, it seems plausible that an innate stimulus that leads to increased TNF-α, could help elicit a cascade of events associated with acute response [3]. Rather than the increase of TNF-α *per se*, these stimuli could produce a series of interconnected events, of which TNF-α upregulation might be one of the consequences. For example, the engagement of toll-like-receptors (TLRs) by the endogenous danger-associated molecules (the rise of which can be caused by the intervention itself or by the ischemic-reperfusion injury) [97,143], may lead to NF-kB (nuclear factor kappa B) activation and transcription of NF-kB induced genes, including TNF-α [144]. TNF-α is a potent activator of NF-kB, thereby amplifying a positive feedback mechanism. Moreover, NF-kB, by inducing transcription of CXCR3 and CCR5 ligands [144], could trigger and sustain the IFN-γ cascade by promoting the migration of IFN-γ-producing Th1 cells, cytotoxic T cells, and NK cells. Concurrently, the activation of TLRs on antigen presenting cells (APCs) could also enhance antigen presentation and induce upregulation of co-stimulatory molecules, promoting adaptive responses and recruiting CTLs [145,146], with further amplification of the immune response.

In allograft rejection, the continuous and abundant availability of antigens from the surface of donor cells, and the interaction with T and possibly with B cells (directly through interaction of B cell receptor and MHCs) cause a labile condition particularly vulnerable to being switched to a destructive acute response. Thus, whether this condition is sufficient *per se *to determine an acute response (according to the self non-self model) or needs to be prompted (in accordance with the danger model), is object of ongoing debate [137,147].

In conclusion, we could hypothesize that both innate and adaptive mechanisms synergize in generating/sustaining the immune response. Indeed, the dual presence of such strong stimuli leads almost inevitably to a progressive destructive response, thereby requiring lifelong immunosuppression, with the exception of the rare cases of spontaneous tolerance [103,128,148].

#### Complement

Complement is the archetypal innate defense mechanism and provides a vital link between innate and adaptive functions [149-151]. Briefly, the central event in complement activation is the proteolysis of C3 (activated by antibodies or microbial cell surfaces) to generate biologically active products that lead to the formation of membrane attack complexes that result in the activation of granulocytes and cell lysis [149-151]. The majority of complement is synthesized in the liver; however, local sources of complement include endothelial cells, macrophages, neutrophils, and epithelial cells (particularly renal tubular epithelial cells). The molecular pathways that lead to the activation of complement transcription during the alloresponse are not completely clear, but activation of the NF-kB pathway has been suggested to be a potential stimulus for local C3 production [152,153]. It has been proposed that C3 could also be responsible for the Th1 response observed during allorejection, directly by sustaining Th1 development [154], or indirectly by inhibiting Th2 polarization [155]. Priming C3 deficient mice with dendritic cells led to delayed skin allograft rejection. Additionally, complement can activate B cells and initiate humoral responses [156]. In kidney transplantation in animal models, it has been shown that local renal C3 production leads to faster allograft rejection [157,158]. However, opposing results were reached in three independent studies analyzing liver transplantation in animal models. In these cases, an association between overexpression of C3 and tolerance was found [159-161]. Thus, at least in animal models, it is possible to hypothesize the existence of diverse regulatory mechanisms in different organs.

The presence of C4d (a C4 split product) by immunohistochemical staining is a feature associated with antibody-mediated rejection since it can activate the classical pathway of complement. Since the majority of circulating complement is produced by the liver, complement is not typically detected by microarray analysis. Thus, detection of complement transcripts during acute allograft rejection by gene expression suggests local production within the graft.

C3 and/or other complement components (C1 and C4) have been associated with acute allograft rejection in several microarray studies conducted in renal [23,21], liver [15], heart [19,25], and lung [17] transplants. Currently, interest in the role of complement in the regulation of the alloresponse is rising [149,150,162]. In a recent study conducted by Naesens et al. (Stanford group [162]), the authors observed upregulation of complement genes before transplantation in deceased donor kidney biopsies compared to living donors. In the same publication, the authors reported a significant overexpression of genes involved in the complement cascade (including C1 and C3) when comparing 32 acute rejection samples to 20 non-rejection samples obtained from pediatric kidney recipients [162].

### IL-10

Contrary to the popular belief that IL-10 is principally an anti-inflammatory cytokine, the IL-10 pathway is frequently described as upregulated during acute allograft rejection in kidney and liver transplants in humans [14,23,26] (Table 1).

Although the canonical effects of IL-10 are regulatory and function in the termination of inflammatory processes [163], this cytokine cannot merely be classified as anti-inflammatory, due to its pleiotropic ability to both positively and negatively influence the function of innate and adaptive immunity in pre-clinical models [164-166]. In humans, intravenous administration of recombinant IL-10 produces pro-inflammatory effects by enhancing the release of IFN-γ, TNF-α, and IL-1, and appears to induce the activation of CTLs and NK cells, as reflected by increased plasma levels of granzyme-B [167,168]. Interestingly, high levels of serum IL-10 were associated with anti-tumor response in a clinical trial involving metastatic melanoma patients treated with immunochemotherapy (i.e., bevacizumab and fotemustine) [169]. In human monocyte lineage cells, IL-10 increases the expression of TLRs, which might sensitize these cells to 'danger signal' mediators. This suggests that IL-10 plays a key role in the early phases of the acute immune response. Systemic administration of IL-10 exacerbates alloreactions in murine models [170,171], and, accordingly, the administration of anti-IL-10 monoclonal antibody prolongs graft survival [172]. In addition, by inhibiting APC maturation and postponing their migration to lymph nodes, this cytokine may lead to more efficient antigen loading, and might activate locally adaptive effectors [164-166]. In humans, post-transplant levels of IL-10 [173] and a specific IL-10 polymorphism [174] were associated with risk of acute rejection in kidney transplants.

The evidence provided supports IL-10 involvement in tumor rejection and allograft rejection in humans, and suggests that this cytokine defies its reputation of having solely anti-inflammatory properties.

#### IRF-1 and STAT-1

**Figure 2:**
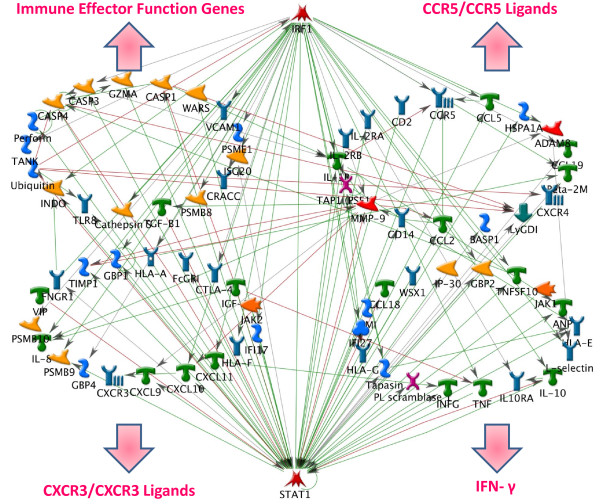
**Transcription Regulatory Network Analysis according to MetaCore algorithms**. This figure shows possible genes regulated by STAT-1 and IRF-1. The gene list uploaded represents the *key gene list *(Table 1).

By using MetaCore algorithms, IRF-1 and STAT-1 were predicted to be regulators of several of the key transcripts after analysis of our key genes list extracted from microarray studies (Figure 2). IRF-1 is an inducible IFN-γ transcription factor and it is transcribed in response to IFN-γ via STAT-1 [54,175]. This transcription factor could mediate the upregulation of several gene/gene pathways during acute allograft rejection, as shown in Figure 2. Genes upregulated by IRF-1 include pro-inflammatory cytokines (e.g. TNF-α [176]), chemokines (e.g. CXCL10 [66,67], CCL5 [68]), and MHC class I and class II molecules [54,177]. It could also drive the synthesis of IL-10 RA [66]. Another important pro-inflammatory function of this gene is the induction of IL-12 [178] and IL-15 [179] with consequent enhancement of the IFN-γ cascade.

IRF-1 has been better described with relation to tumor rejection. In a study conducted in melanoma patients by Wang et al. [2], IRF-1 was the most significantly and consistently upregulated transcript in metastatic melanoma lesions undergoing clinical regression after the systemic administration of high-dose interleukin-2. IRF-1 appeared to play a central role in orchestrating the immune response, generating the switch from chronic to acute inflammation in this as well as several subsequent studies [3,4].

Regarding the allograft, although statistical algorithms recognize IRF-1 as one of the main transcription factors that regulate genes involved in acute allograft rejection, it should be noted that its overexpression *per se *has not yet been identified as relevant according to human microarray studies. Thus, these data must be interpreted cautiously. Nevertheless, STAT-1 has been massively described as upregulated during acute allograft rejection (see Table 1), suggesting the regulation of IRF-1 through the IFN-γ/STAT-1 pathway as a plausible mechanism. In a recent mouse liver transplant model microarray study, IRF-1 was one of the two genes overexpressed both in leukocytes and intragraft during acute cellular rejection (GBP2 was the other gene, also an IFN-γ inducible gene) [180]. Accordingly, studies have reported an association between IRF-1 and acute cellular rejection in heart transplant models [181,182]. On the other hand, other groups have reported STAT-1/IRF-1 pathway to be upregulated in tolerant models [159,160]. In order to explain these findings, investigators proposed the induction of T cell apoptosis by IFN-γ signaling [159]: the transcripts STAT-1 and IRF-1 were also found to be involved in the induction of apoptosis via a caspase-mediated mechanism [183] (Figure 2). Thus, it is likely that IRF-1 plays a different role according to the independent co-activation of different pathways, which can greatly differ from cell to cell but can also vary with changes in the surrounding environment [183].

Although IRF-1 seems to be regulated primarily by IFN-γ signaling [175], *in vivo *and *in vitro *observations suggested that IRF-1 regulation does not necessarily require the interaction of this cytokine. Indeed, IRF-1 has been observed in response to IL-2 stimulation *in vitro *[184] and in the absence of interferon upregulation in animal models [100]. In addition, IRF-1^-/-^mice have defects not observed in IFN-γ or IFN-γ- receptor ^-/- ^animals, (such as alterations in CD8+ T cells and thymocyte development), supporting the existence of an IFN-γ-independent activation pathway of IRF-1, [54,185]. *Vice versa*, even supposing a central role for this protein in the induction of pro-inflammatory mediators, a recent microarray study in heart transplanted mice suggested that IRF-1 functions could be bypassed by other mediators [186]. That same study showed that the expression profile of the allograft from IRF-1^-/- ^mice and wild type mice were nearly identical to each other and very different from the profile of isograft control.

### Comparative analyses

Despite discrepancies among different studies, cross-comparison of datasets has been remarkably revealing [25,26], probably because of the highly conserved molecular patterns associated with immune-mediated tissue destruction. The first comparative analysis was performed by Morgun et al. [25] who, after identifying a gene set predictive of acute-rejection in a series of heart allograft recipients, analyzed the data from two published studies on kidney (Stanford dataset [187] and Cleveland dataset [23]) and lung (Minneapolis dataset [17]) transplants. The authors observed a striking agreement with the histological diagnosis of the three studies. The predictive accuracy of the gene set obtained from studying hearts was close to 95% in kidney and lung acute rejection illustrating the similarity of activated pathways from different rejected organs. Similar to observations in renal transplants [22], B cell transcripts (immunoglobulins) were among the most upregulated, suggesting that B cells may also have a local effect in heart rejection. Another interesting finding was the similar pattern of immune-response-related gene expression (antigen presentation, innate immunity, chemotaxis, immunoglobulins and cytokines) among samples with diagnosis of acute rejection versus infection. Here, the gene expression pattern of transplant recipients who underwent rejection was similar to that of patients with Trypanosoma cruzi infection (which represents a frequent cause of chronic heart failure and consequent need for heart transplant in Latin America) [25]. The similarities in inflammatory/immune expression patterns between acute rejection and infection have also been described by Sarwal et al. [22].

By utilizing an established protein prediction model for discovering serum biomarkers of disease, (Integrated RNA Data Driven Proteomics (IRDDP)), Chen et al. [27] applied this model to cross-organ acute allograft rejection datasets. In this analysis, three existing gene expression datasets were analyzed to identify candidate serum protein biomarkers. Evaluation of the three datasets revealed 45 genes commonly differentially expressed in acute allograft rejection (see Table 1). The datasets were extracted from GEO and were derived from microarray studies conducted on pediatric renal, adult renal, and adult cardiac human tissue biopsies during acute allograft rejection. Interestingly, by applying this protein biomarker prediction model, this data guided the investigators to discover three serum protein biomarkers, PECAM1, CXCL9, and CD44, that could distinguish acute rejection from stable allograft function. Notably, since gene expression data was compared in heart and kidney samples, it reinforces the principal that common molecular mechanisms exist in acute allograft rejection across different organs.

Another comparative analysis was conducted by Saint-Mezard et al. [26] analyzing three datasets from human renal acute allograft rejection microarray studies. These authors compared their own data, which consisted of human and non-human primate kidney acute rejection biopsy specimens, to the Stanford [22] and Cleveland [23] datasets. By doing so, the authors analyzed 36 acute rejection samples, identifying 70 genes that were upregulated during acute allograft rejection. Importantly, they successfully validated their findings by using 143 microarrays from the Edmonton dataset [31].

**Figure 3:**
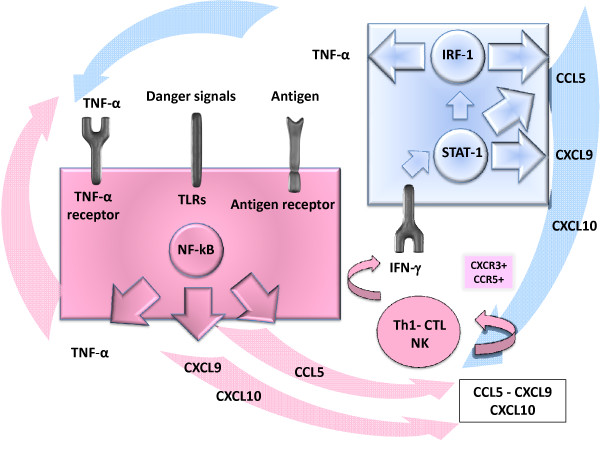
**Possible mechanism of reciprocal enhancement between innate and adaptive immunity, through NF-kB and STAT-1/IRF-1 pathway**. This sketch is built according to genes often described as upregulated during acute allograft rejection in human studies. NF-kB can be activated by a variety of inflammatory stimuli. For example, the engagement of toll-like receptors (TLRs) by the endogenous danger-associated molecules may lead to NF-kB activation and transcription of NF-kB induced genes, including TNF-α. TNF-α is a potent activator of NF-kB, thus forming an amplifying feed-forward loop. Indeed, NF-kB, through inducing transcription of CXCR3 and CCR5 ligands (e.g. CXCL9, -10 and CCL5 respectively), engages Th1 cells, CTLs and NK cells since all express CXCR3 and CCR5. These cells in turn produce IFN-γ with consequent activation of the STAT-1/IRF-1 pathway leading to further production of chemoattractants (CCR5 and CXCR3 ligands) with amplification of the IFN-γ response. IRF-1 can also induce TNF-α production, with further amplification of the loop.

Using GeneGo MetaCore algorithms (a web-based suite for functional analysis of experimental data http://www.genego.com) STAT-1, Interferon Regulatory Factor (IRF-1), Nuclear Factor Kappa B (NF-kB), and PU.1 (a transcription factor involved in the in the development of myeloid and lymphoid cells [188]) were identified as the main transcription factors that regulate the 70 genes consistently represented during kidney acute rejection in according to the Saint-Mezard comparative analysis [26]. The relationship among different protein-protein interactions, activation of transcription factors, and functional response is often difficult to establish because of its complexity and due to the incompletely understood association among signaling pathways. In simple terms, during the alloresponse, NF-kB represents a constitutive activation of innate immunity (e.g. TNF-α pathway), and IRF-1 superimposes a switch toward adaptive immunity (through the IFN-γ pathway). As described previously, these two pathways can amplify each other, and can also collaborate in inducing the transcription of common genes. For example, IFN-γ (through IRF-1) and TNF-α (through NF-kB) can synergize in promoting the overexpression of common genes such as CXCR3 ligands [67] and CCR5 ligands [144]. Figure 3 summarizes a likely reciprocal enhancement of function between the NF-kB and the STAT-1/IRF-1 pathways during allograft rejection. Beyond the function of master regulator of innate immunity, NF-kB is also important in driving the adaptive response. In fact, it plays a key role in IL-2 and TCR signaling, and in the regulation of immunoglobulin production [152]. It should be noted that most of the drugs effective in the treatment and/or prevention of acute allograft rejection (e.g. glucocorticoids, cyclosporine, and tacrolimus), interact with NF-kB pathway, and result in reduced production of several cytokines such as IL-2 and TNF-α [152]. Accordingly, NF-kB activity impairment leads to an attenuation of acute rejection in heart [189-191], lung [192] and skin [193] in animal models.

### Metzincins and Related Genes

Recently, attention has been brought to the role of the metzincins (a superfamily of endopeptide cleaving extracellular matrix proteins implicated in remodeling and modulation of cell signaling) in acute allograft rejection. In the Rodder meta-analysis, expression of Metzincins and Related Genes (MARGs) were analyzed from four separate microarray study databases to characterize markers of acute rejection in renal transplantation [22,23,29,31], revealing MMP7 and TIMP1 as the most highly upregulated genes [29]. Interestingly, MMP9, TIMP1, and ADAM genes have also been noted to be associated with liver and heart acute allograft rejection [14,15,25] as listed in Table 1 of this review. Further, MMP9 and MMP2 were also described to be upregulated in a case report of small bowel acute rejection profiled by microarray. Of note, IFN-γ was also upregulated in this case report emphasizing the similarity cross-organ in acute allograft rejection [20].

## Conclusions

High-throughput gene expression profiling has emerged as a powerful and reliable tool in investigating immune response *in vivo *in humans [11,12]. Bypassing the traditional hypothesis-driven approach, microarray studies have revealed unsuspected mechanisms that mediate the balance between rejection and tolerance.

The pathways thought to be central during acute allograft rejection have been described in this review. Most of the pathways analyzed (IFN-γ/STAT-1/IRF-1 path, CXCR3/CXCR3 ligands path, CCR5/CCR5 ligands path, and IEFs path) have also been associated with other immune-mediated processes, strengthening the concept that there are common convergent molecular mechanisms in tissue specific destruction, as described by the Immunologic Constant of Rejection [3,194-198]. Even if the pathways analyzed are consistently observed in humans, previous experiments in animal models failed to demonstrate them as necessary or sufficient for the development of rejection, in concordance with the high redundancy of mammalian immune system [54,82,83]. Moreover, some of the genes associated with acute rejection also seem to play a role in tolerance models (e.g.STAT-1/IRF-1 [159,160]), stressing the pleiotropism of such molecules, as well as illustrating the complexity of these networks and the necessity of investigating immune-response mechanisms *in vivo *in humans. Despite the wide-ranging observations at molecular level which could be significantly influenced by multiple factors including sample collection time, sample type, sample handling and storage conditions, patient physiological condition, coexisting pathological conditions, environmental factors and genetic predisposition, distinct molecular patterns associated with tissue destruction have been revealed and summarized in this review.

In conclusion, the purpose of this review was to contribute to the understanding of *how *tissue specific destruction occurs. Understanding *why *this occurs is one of the most challenging and intriguing questions facing modern human immunology.

## Competing interests

The authors declare that they have no competing interests.

## Authors' contributions

DB and TLS performed data extraction from the available literature and prepared the manuscript collaboratively with input and review by all co-authors. FMM and EW reviewed data extraction and literature research and revised the manuscript for important intellectual contents. All authors read and approved the final manuscript.

## Supplementary Material

Additional file 1 **supplemental data extraction information**. supplemental information for key gene selection used for Table 1, IPA and Meta Core analysis. Comprehensive lists of relevant upregulated genes, in according with the original publication, are reported for the following studies: Akalin et al, Tannapfel et al, Sreekumar et al, Karason et al (genes most frequently upregulated during the rejection episode and returned to baseline levels with its resolution), Reeve et al (genes most frequently represented in the predictive analysis for microarrays classifier), Saint-Mezard et al. Morgun et al: we reported the upregulated genes selected from the list of 98 genes belonged to the first predictor set that discriminate acute cardiac, renal and lung rejection from non rejection. CCL5 belonged to the second prediction set. Sarwal et al: we selected key immune genes from the list of genes upregulated in three different subtypes of acute allograft rejection (see also Mansfield et al. 2004 and Weintraub et al 2006). Gimino et al and Lande et al: we selected key genes from a list of genes reported as upregulated during acute rejection according to the first (Gimino et al) and second (Lande et al) analyses. Flechner et al: we selected key genes a list of genes upregulated in acute rejection samples compared to samples without diagnosis of rejection. Others upregulated genes included in the original list were: Morgun et al: Homo sapiens cDNA FLJ10266 fis, clone HEMBB1001024; Homo sapiens cDNA FLJ10580 fis, clone NT2RP2003533, mRNA sequence; Homo sapiens cDNA FLJ10981 fis, clone PLACE1001610; Homo sapiens mRNA, cDNA DKFZp434P1019; Homo sapiens mRNA; cDNA DKFZp564P073; Homo sapiens mRNA; cDNA DKFZp586H0718; Homo sapiens mRNA; cDNA DKFZp761G0924; Homo sapiens mRNA; cDNA DKFZp761P221; DKFZP434B033; Unknown (protein for IMAGE:4251653) [Homo sapiens], mRNA sequence; Unnamed protein product [Homo sapiens]. Karason: Homo sapiens Alu repeat (LNXI) mRNA sequence. Reeve et al: affymetrix id 235529_at and 238725_at. The 14 genes selected in the Inkinen et al study were those genes highly upregulated in AR vs NR control. Morgun et al: we reported the upregulated genes selected from the list of 98 genes belonged to the first classifier that discriminate acute cardiac rejection vs non rejection and immune-related genes selected from the second and third classifier (130 and 188 genes respectively): the three classifier also discriminated rejection and non rejection lung and kidney samples. Asaoka et al analyzed biopsies from 21 liver transplant recipients with recurrent HCV (RHC). Analysis compared 9 with AR + RHC versus 13 with RHC only (control). Genes shown in this table selected from the network classified as "Cell death, hematological disease, and immunological disease" via IPA. Click here for file
