# Supplementary material for: Gene expression profiling in acute allograft rejection: challenging the immunologic constant of rejection hypothesis
Source: J Transl Med. 2011 Oct 12;9:174. doi: 10.1186/1479-5876-9-174 (PMC3213224; doi:10.1186/1479-5876-9-174)
Supplement: Additional file 1 — supplemental data extraction information. supplemental information for key gene selection used for Table 1, IPA and Meta Core analysis. Comprehensive lists of relevant upregulated genes, in according with the original publication, are reported for the following studies: Akalin et al, Tannapfel et al, Sreekumar et al, Karason et al (genes most frequently upregulated during the rejection episode and returned to baseline levels with its resolution), Reeve et al (genes most frequently represented in the predictive analysis for microarrays classifier), Saint-Mezard et al. Morgun et al: we reported the upregulated genes selected from the list of 98 genes belonged to the first predictor set that discriminate acute cardiac, renal and lung rejection from non rejection. CCL5 belonged to the second prediction set. Sarwal et al: we selected key immune genes from the list of genes upregulated in three different subtypes of acute allograft rejection (see also Mansfield et al. 2004 and Weintraub et al 2006). Gimino et al and Lande et al: we selected key genes from a list of genes reported as upregulated during acute rejection according to the first (Gimino et al) and second (Lande et al) analyses. Flechner et al: we selected key genes a list of genes upregulated in acute rejection samples compared to samples without diagnosis of rejection. Others upregulated genes included in the original list were: Morgun et al: Homo sapiens cDNA FLJ10266 fis, clone HEMBB1001024; Homo sapiens cDNA FLJ10580 fis, clone NT2RP2003533, mRNA sequence; Homo sapiens cDNA FLJ10981 fis, clone PLACE1001610; Homo sapiens mRNA, cDNA DKFZp434P1019; Homo sapiens mRNA; cDNA DKFZp564P073; Homo sapiens mRNA; cDNA DKFZp586H0718; Homo sapiens mRNA; cDNA DKFZp761G0924; Homo sapiens mRNA; cDNA DKFZp761P221; DKFZP434B033; Unknown (protein for IMAGE:4251653) [Homo sapiens], mRNA sequence; Unnamed protein product [Homo sapiens]. Karason: Homo sapiens Alu repeat (LNXI) mRNA sequence. Reeve et al: affymetrix id 235529_at a [file 1479-5876-9-174-S1.DOC]

**S1 Supplemental information for key gene selection used for Table 1, IPA and Meta Core analysis.**

Comprehensive lists of relevant upregulated genes, in according with the original publication, are reported for the following studies: *Akalin et al, Tannapfel et al, Sreekumar et al, Karason et al* (genes most frequently upregulated during the rejection episode and returned to baseline levels with its resolution), *Reeve et al* (genes most frequently represented in the predictive analysis for microarrays classifier), *Saint-Mezard et al*. *Morgun et al:* we reported the upregulated genes selected from the list of 98 genes belonged to the first predictor set that discriminate acute cardiac, renal and lung rejection from non rejection. CCL5 belonged to the second prediction set. *Sarwal et al*: we selected key immune genes from the list of genes upregulated in three different subtypes of acute allograft rejection (see also Mansfield et al. 2004 and Weintraub et al 2006). *Gimino et al* and *Lande et al:* we selected key genes from a list of genes reported as upregulated during acute rejection according to the first (Gimino et al) and second (Lande et al) analyses. *Flechner et al:* we selected key genes a list of genes upregulated in acute rejection samples compared to samples without diagnosis of rejection.

Others upregulated genes included in the original list were: *Morgun et al*: Homo sapiens cDNA FLJ10266 fis, clone HEMBB1001024; Homo sapiens cDNA FLJ10580 fis, clone NT2RP2003533, mRNA sequence; Homo sapiens cDNA FLJ10981 fis, clone PLACE1001610; Homo sapiens mRNA, cDNA DKFZp434P1019; Homo sapiens mRNA; cDNA DKFZp564P073; Homo sapiens mRNA; cDNA DKFZp586H0718; Homo sapiens mRNA; cDNA DKFZp761G0924; Homo sapiens mRNA; cDNA DKFZp761P221; DKFZP434B033; Unknown (protein for IMAGE:4251653) [Homo sapiens], mRNA sequence; Unnamed protein product [Homo sapiens]. *Karason:* Homo sapiens Alu repeat (LNXI) mRNA sequence. Reeve et al: affymetrix id 235529_at and 238725_at.

The 14 genes selected in the *Inkinen* et al study were those genes highly upregulated in AR vs NR control

*Morgun et al:* we reported the upregulated genes selected from the list of 98 genes belonged to the first classifier that discriminate acute cardiac rejection vs non rejection and immune-related genes selected from the second and third classifier (130 and 188 genes respectively): the three classifier also discriminated rejection and non rejection lung and kidney samples .

*Asaoka et al* analyzed biopsies from 21 liver transplant recipients with recurrent HCV (RHC). Analysis compared 9 with AR + RHC versus 13 with RHC only (control). Genes shown in this table selected from the network classified as “Cell death, hematological disease, and immunological disease” via IPA.
